# Identifying dietary patterns and their association with oral health behaviors in young children: a parent-based cross-sectional study in Lithuania

**DOI:** 10.3389/froh.2026.1844389

**Published:** 2026-06-15

**Authors:** Yvonne A. B. Buunk-Werkhoven, Rasa Tamulienė, Evelina Dailidaitė, Daiva Mačiulienė

**Affiliations:** 1 Faculty of Medicine, Oral Health Department, Kauno Kolegija Higher Education Institution, Kaunas, Lithuania; 2 Faculty of Medicine, Department of Medical Technologies and Dietetics, Kauno Kolegija Higher Education Institution, Kaunas, Lithuania

**Keywords:** health promotion, Lithuania, oral health and consumption habits, parental influence, pre- and primary school children

## Abstract

**Background:**

Children’s oral health and nutrition are interconnected, jointly influencing growth, oral development, and disease prevention such as caries. Despite this connection, they are often addressed separately in research and practice, underscoring the need for validated parental assessment tools that support integrated and interdisciplinary prevention strategies.

**Methods:**

This cross-sectional study examined underlying dietary patterns among 420 children aged 5–12 years in Kaunas, Lithuania, based on parental reports. Data were collected as part of a broader investigation of oral health behavior (OHB) and knowledge. Children's consumption habits were assessed using 17 structured dietary items, which were further used to adapt nutritional assessment subscales. Ethical approval was obtained from Kaunas Kolegija HEI. Exploratory factor analysis (EFA) was conducted to identify underlying dietary dimensions, and associations with sociodemographic variables and OHB were examined using correlation and regression analyses.

**Results:**

EFA revealed a three-factor model explaining 40.30% of total variance, comprising: “recommended healthy foods” (e.g., fruits, vegetables), “inappropriate drinks and snacks” (e.g., sugary beverages), and “dairy products.” Dietary measures were not associated with parents' age, gender, or self-perceived nutrition knowledge. Parents' education type was positively associated with “dietary” and “recommended healthy foods”, while children's age showed negative associations with these variables, indicating less healthy dietary patterns in older children. OHB was positively associated with “recommended healthy foods”, “dietary”, and fruit and vegetable intake, but negatively associated with inappropriate drinks and snacks. Regression analysis revealed that consumption of “recommended healthy foods” and “parental success in ensuring healthy eating” as significant independent predictors of optimal OHB.

**Conclusions:**

These findings provide empirical support a multidimensional, parent-based dietary assessment framework that captures distinct eating patterns relevant to oral health. Integrating such tools into oral health promotion may enhance early prevention strategies by linking dietary behaviors with oral hygiene practices in a structured and evidence-based manner.

## Introduction

The world has become more modern and fast-paced, and our consumption habits have changed dramatically. People now consume more high-calorie, nutritionally poor foods, such as sweets, salty snacks, sugary drinks, and fast food. Diets high in sugar, fat, processed meat, and salt are widespread, and these dietary patterns lead to increasing health problems such as obesity, diabetes, and other chronic diseases (1). Various multiple nutrition-related concepts are used in the literature, and in this study, “consumption” is used and interpreted as a descriptive term referring to the intake or consumption of food and beverages to satisfy needs or desires. A healthy diet is essential for human growth, development, and livelihood and is therefore also important for oral health (2). In particular, the frequency of added sugar consumption is strongly associated with tooth decay, and several demographic and lifestyle characteristics; among children who consume more foods and drinks with added sugar more often have a higher risk of tooth decay (3, 4). Healthy and attractive teeth are highly valued, due to their contribution to appearance and social acceptance (5).

Nutrition and diet are important determinants of oral health and diseases (e.g., caries). This relationship is complex and reciprocal, and there is a growing awareness that dental problems themselves can negatively impact food choices, food consumption, and nutritional status (6). Research on social determinants and patterns of nutrition and oral health shows that the environment, income, culture, and access to healthy food all influence dietary behaviors and oral health outcomes (7). According to current healthy eating recommendations, vegetables and fruits, whole grain products, as well as nuts and fish should dominate a healthy diet (1, 8, 9).

To ensure optimal (oral and nutritional) health, it is crucial that children develop proper oral hygiene habits and eating and snacking habits from an early age, preferably with the support of their parents or caregivers (10). Health behaviors established during childhood, especially as children (ages of 6–12 years) lose primary teeth and develop permanent dentition. This is an important period when healthy lifestyle and dietary habits are formed immediate and long-term health outcomes. Poor self-care, including oral hygiene and eating habits, developed in childhood can persist into adulthood. For instance, early dietary changes have been shown to enhance health and reduce the risk of developing diseases later in life. So, it is essential that children receive accurate information about health-promoting nutrition, oral hygiene behavior and its significance for the body (7–11). Pre- and primary school children gain increasing independence in their daily living activities (ADLs), including oral self-care routines and dietary choices. Therefore, it is crucial to guide parents on appropriate oral hygiene behavior and eating habits (10–13). Oral hygiene and eating habits influence not only the optimal physical and mental development of children, but early adoption healthy practices has been shown to reduce the risk of chronic diseases and improve their health in adulthood (8).

Despite growing awareness of the importance of these health behaviors, recent studies show that children's lifestyles, tooth brushing, and diet in Lithuania and other countries are not optimal: parents of children have sufficient oral health knowledge, but this knowledge is not consistently translated into effective practices; their brushing habits are suboptimal (10). Moreover, children attending general education schools in Lithuania don't eat enough vegetables, fruits, and berries, and they opt for sugary snacks, eat fast food, and don't follow a consistent eating pattern; younger children having breakfast and eating hot meals at school more frequently than older children, and younger children also consuming more vegetables than older ones (11, 14). In addition, the environment: family, school and political decisions, plays an important role in learning health skills (1, 10, 15). The example that parents set, or their role as primary role model, is one of the most important factors. Therefore, when raising children, it is necessary not to forget to improve parents' oral care and eating habits, increase their health knowledge and reduce the availability of unhealthy foods and snacks at home. Parental behavior determines the available oral care and food products, brushing and eating habits, traditions and the family's attitude towards (oral) health and nutrition. Efforts to reduce the availability of unhealthy foods at home, promote consistent oral hygiene routines, and enhance parental engagement are critical components of effective interventions (1, 10, 14, 16). These findings highlight a discrepancy between knowledge and behavior, suggesting that awareness alone is insufficient to bring about meaningful change.

Although existing literature clearly links dietary patterns, particularly frequent consumption of added sugars, to poor oral health outcomes in children, most studies focus on describing behaviors and associated risk factors rather than translating this knowledge into practical, age-appropriate interventions. Furthermore, while the influence of social, environmental, and cultural determinants on both nutrition and oral health is well established, there is a lack of integrated tools or frameworks specifically designed to guide effective preventive strategies in younger populations. To fill these gaps, an attempt is made to provide a more comprehensive insight into children's health behavior, with a specific focus on the interplay between nutrition, oral hygiene, and parental influence, thereby contributing to the development of more effective health promotion strategies.

From social psychology perspective, Kurt Lewin (1951, p. 169) stated “There is nothing so practical as a good theory”. This implies that the application of theoretical models in human and social sciences and the use of scientific evidence can contribute to understanding and potentially solving practical problems. Here, the use of the Health Belief Model (HBM) (17); helps explain why people make certain health-related choices. The model clarifies how a person's beliefs, attitudes, and risk perceptions influence their eating habits and management of (oral) health behavior. Based on this model, preschoolers and elementary school children can be approached as “targets of change” by empowering their parents as “agents of change” (18). Ultimately, the goal is to design effective, theoretically sound health promotion interventions grounded in widely applied health behavior theories, where a fun and active learning process plays a key role in developing healthy oral hygiene and eating skills (10, 19). In this practical project, this translates into applying the Problem-Analyze-Test-Help-Success (PATHS) model (20), thereby contributing in an effective and efficient way to disease prevention and the improvement of overall well-being.

### Overview of present research

The purpose of this study is to investigate the relationship between nutrition and oral health in children aged 5 to 12 and, by integrating the insights, to explore how oral health and nutrition professionals can align their recommendations to improve both eating habits and oral health within the family context. Moving beyond purely descriptive findings, the study aims to develop a structured, evidence-based intervention that simultaneously targets dietary behaviors and oral health practices in preschool and primary school children.

## Materials and methods

### Study design and setting

This cross-sectional study was conducted among parents of pre-school and primary school children and was administered to a sample of the Lithuanian population from March 13 to 28, 2024. For the complete data collection an online survey with 33 questions in Lithuanian via the LimeSurvey platform was used (10, 14, 21, 22). A representative from the Public Health Service sent an invitation to participate in the survey to public health specialists working in schools in Kaunas that implement preschool and primary education programs. These specialists distributed the invitation, including a link to the survey, via the schools' usual internal communication channels (such as email and electronic diary entries) to all parents of children aged 5 to 12.

### Study size and participants

A non-probabilistic sampling method was applied, with a minimum required sample of 374 parents to ensure 95% confidence. The final sample comprised 420 parents (M^age^ = 37.3, SD = 4.6) of preschool and primary school children in Kaunas. As in previous studies (10, 14, 22), ethical approval was granted by the Applied Scientific Research Ethics Compliance Committee of Kaunas Kolegija HEI (No. 13–14). The study was conducted in collaboration with the Kaunas Public Health Bureau in accordance with the Helsinki Declaration. Participation was voluntary, and anonymity and confidentiality were ensured. Informed consent was obtained from all participants prior to participation in the study. At the beginning of the online questionnaire, participants were shown an introductory section explaining the purpose and content of the study, after which they were asked for consent to participate. Consent was granted electronically (i.e., written informed consent) by completing the questionnaire. A total of 532 individuals opened and reviewed the questionnaire; of these, 11 respondents after familiarizing themselves with the aim and content of the study, did not agree to participate. In total, 521 respondents proceeded to the next stage and completed the questionnaire. After reviewing the collected data, it was found that 20 respondents had completed the questionnaire incorrectly (e.g., indicated the ages of multiple children instead of one; provided the child's age instead of the parent's age, etc.). Therefore, 501 responses were deemed suitable for further analysis. Further data screening revealed that 81 respondents did not meet the inclusion criteria, as they indicated raising children younger than five or older than twelve years. These responses were excluded from further analysis. The final data analysis was conducted using responses from 420 participants.

### Questionnaire

In December 2023–February 2024, the questionnaire was compiled, verified, and translated directly from English into Lithuanian as its mother tongue by a native Lithuanian speaker of Lithuanian descent, using a double translation method and collaborative discussions by experts to tailor the content for pre-school and primary school children. Using the Delphi method, i.e., a structured process involving a multidisciplinary expert group the indices were adapted and culturally updated from the parents' perspective. The adapted versions were reviewed and the indices were pilot-tested with parents, and their feedback informed the final adjustments, resulting in completed questionnaire (22). The questionnaire introduction explained the study purpose, data use, and contact details. No personal identifiers were collected, and data were analyzed only in aggregate to ensure confidentiality. The first page of the 33-item questionnaire (10) included an introductory section describing the purpose of the study, and covered demographics and assessed parents' knowledge, behaviors, and motivational practices related to children's dietary habits, including a 17 items, a series of questions about dietary and oral health using multiple-choice items and Likert-type scales. Some questions, for instance concerning breakfast, fruits or vegetables and drinks refer to the patterns and behaviors related to how, when, and what the children eat.

*Dietary* was measured using 17 items, a series of questions that has been used previously in this composition and originates from a cross-sectional study Lithuanian COSI study (14, 23); the reliability was measured with Cronbach's alpha = .66, and with an alternative measure of reliability, Guttman's lambda 2 = .69. Parents evaluating their children's dietary patterns. Some examples are, “Fresh fruit (excluding juice or dried fruit); How often does your child eat and drink these foods and beverages during a typical week?”, “Sugary soft drinks (Coca-Cola, lemonade, etc.); How often does your child eat or drink the following foods and beverages during a typical week?”, “Cheese (hard cheese, cheese sticks, etc.); How often does your child eat or drink these foods and beverages during a typical week?”, “Sweet breakfast cereals; How often does your child eat or drink these foods and beverages during a typical week?” and “Legumes (beans, peas, lentils, etc.); How often does your child eat or drink these foods and beverages during a typical week?” Responses were scored on a six-point scale, i.e., “never” (0), “less than once a week” (1), “1–3 days/week” (2), “4–6 days/week” (3), “once a day” (4), and 'several times a day’ (5). The sum Dietary score on this measurement could range from 0 to 102. A high sum score indicated a greater overall consumption and a stronger presence of the measured eating habits.

*Oral hygiene-related behavior* was assessed using the for Lithuania adapted Oral Hygiene Behavior index (OHB) (10, 22); for preschool and primary school children from parents perspective, retaining all original criteria except interdental care (24). This 7-item index, i.e., (1) the frequency of dental brushing; 2) the time of day when teeth are cleaned; 3) pressure forces when brushing teeth; 4) the duration of tooth brushing; 5) teeth-brushing movements; 6) type of toothpaste; 7) cleaning the tongue, included parental reports on toothbrush type and brushing practices, supported by illustrative images. Each criterion of oral health-related behavior was assigned indicators with a different number of scores. The maximum amount of points that can be scored for the entire index is 15. The higher the score for the indicator, the more optimal the reported oral hygiene-related behavior is. The mean score of this OHB index was 8.6 (SD = 2.0; range 3–14), with a near-normal distribution; it reflects reported OHB rather than a latent construct (22, 24, 25).

*Oral health knowledge* (OHK) was assessed using a 16-item OHK index, adapted for Lithuania, which covers 3 areas and evaluates parents' knowledge and understanding of the children's individual oral hygiene at home (6 items), parents' knowledge of the influence of nutrition on the oral health of children (4 items), and parents' general knowledge of the factors that determine children's oral health (6 items) (10, 22). Items addressed fluoride use, diet, tooth development, and parental involvement. For each of the statements, parents had to choose one option: true or untrue. Each correctly chosen answer option was scored with one score, with a total score of maximal 16. A higher number of points scored indicates a better knowledge of parents about the oral health of children. The mean OHK score was 13.8 (SD = 1.4; range 3–16) (10).

### Statistical analysis

The collected data were downloaded into Excel and in IBM Statistical Package for Social Sciences 29.0 (SPSS Inc., Chicago, IL, USA) files for further data analysis. The calculations included descriptive statistics; the data were subjected to frequency distributions, and means and standard deviations (SDs). For reliability analyses Cronbach's alpha was used, and noteworthy, Guttman's lambda 2 coefficient was used here too as an alternative measure of reliability. Removing items from 17 item Dietary measurement may affect the estimate of reliability, and is justified if there is a rationale for it and face validity is still maintained. Interscale correlation on the Dietary (sub)scales and OHB, OHK was assessed using Pearson's correlations. Also, chi-square and ANOVA tests were used for group comparisons (parental education type, children ages), and calculation of means for quantitative measures. A linear hierarchical regression analysis examined multivariate associations with oral hygiene behavior (OHB) as dependent variable. “Dietary”, “recommended healthy foods”, “inappropriate drinks and snacks” and “the extent to which parents are successful in ensuring their child eats healthily” as predictors were entered simultaneously into the model. Differences were considered statistically significant at *p* < 0.05.

## Results

The sample consisted of 420 parents, predominantly women (94%) with a age range of 24 to 55 years, the majority lived in the city, 10.7% in Kaunas County. The majority had completed higher education (84.5%), while 14% had completed secondary or vocational education, and only a small percentage had completed primary school. Most parents were married (82.6%), and 8.8% were cohabiting. Families had an average of 1.8 children (SD = 0.7), of whom 49% were boys and 50% were girls, and in 4 cases the gender was unknown. Over half had two children (55.2%), a third had one child (31.9%), and a smaller proportion (11.2%) had three or more children. The remaining 1.6% consisted of six families with four children and one family with five children. The average age of the oldest child was 7.35 years (SD = 1.9).

The mean, standard deviation, and range of the total score on the *Dietary measurement* was computed (*M* = 48.42, SD = 6.35, range 29–72), and the distribution of scores was approximately normal. The individual diet score is an indicator of self-reported regular consumption of types and amounts of food and beverages. Assuming a maximum of 102, this means approximately average, which is therefore of little significance. All 17 items (14, 23) combined, or nutrition as a one-dimensional measurement, are theoretically and practically unsuitable. This is because a one-dimensional measurement implies that all items in a scale are assumed to reflect one underlying construct (for example, overall “nutritional quality”) and can therefore be meaningfully added up to a single total score. Here, it is not appropriate to treat the *Dietary measurement* as one-dimensional, because the 17 items likely represent different aspects of the diet, such as high-sugar foods, healthy eating, meal patterns, or beverage choices, which do not necessarily vary together or represent a single concept. When items reflect multiple underlying dimensions, combining them into a single total score can mask important differences, reduce interpretability, and lead to misleading conclusions. Therefore, factor analysis was performed: to identify the different dimensions within the dietary items and to determine whether the measurement might be better subdivided into separate subscales rather than being treated as a single total score.

Based on factor analyses of 17 items, a 5-factor solution was unsatisfactory, but a 3-factor solution comprising “recommended healthy foods” (7 items, i.e., Q1, Q2, Q6, Q7, Q8, Q9, Q17; Cronbach's alpha = .67 and Guttman's lambda 2 = .69), “inappropriate drinks and snacks” (4 items, i.e., Q3, Q13, Q14, Q15; Cronbach's alpha = .60 and Guttman's lambda 2 = .62), and “dairy products” (4 items, i.e., Q4, Q10, Q11, Q12; Cronbach's alpha = .62 and Guttman's lambda 2 = .63) was found to be most satisfactory, and explained 40.30% of the variance. 16 of the 17 items loaded higher than.40 on one of the 3 factors, and not in any other factor. The item “unsweetened breakfast cereals” (Q5) was therefore deleted. The item 'sweet snacks' (Q16) was deleted from the “inappropriate drinks and snacks” subscale, to raise the Cronbach's alpha from.57 to.60 and Guttman's lambda 2 from.59 to.62 (Table 1).

**Table 1:** Dietary measurement (17 items; 14); and a 3-factor solution (*N* = 420).

How often does your child eat and drink these foods and beverages during a typical week?			
Food products	“Recommended healthy foods”	“Inappropriate drinks and snacks”	“Dairy products”
Q1 Fresh fruit (excluding juice or dried fruit)	.58		
Q2 Vegetables (including vegetable soup and stews, excluding potatoes)	.71		
Q3 Sugary soft drinks (Coca-Cola, lemonade, etc.)		.80	
Q4 Sweet breakfast cereals			.60
Q5 Unsweetened breakfast cereals			**.** **32**
Q6 Porridge	.50		
Q7 Meat	.47		
Q8 Fish	.63		
Q9 Eggs dishes	.44		
Q10 Milk			.76
Q11 Cheese (hard cheese, cheese sticks, etc.)			.60
Q12 Yogurt, curd, and other dairy products			.58
Q13 100% fruit juice		.43	
Q14 Soft drinks without sugar, e.g., diet Coca-Cola		.70	
Q15 Salty snacks (potato chips, corn chips, roasted corn, peanuts)		.63	
Q16 Sweet snacks (candies, cookies, pastries, cakes, doughnuts, or pies)	.**80**		
Q17 Sweet snacks (candies, cookies, pastries, cakes, doughnuts, or pies)	.61		

Bold values, Q5 deleted after factor analysis due to insufficient factor loading. Bold values, Q16 deleted from the Inappropriate Drinks and Snacks subscale.

Instead of using a large number of items, it is most adequate and feasible to use subscales, i.e., “recommended healthy foods”, “inappropriate drinks and snacks”, and “dairy products”, comprising 16 items in total. These subscales of 7 and the other two respectively, 4 items are not only statistically, but also theoretically adequate. The mean sum scores of each of these subscales were assessed via calculation of means, and high sum scores indicated a higher consumption of types and amounts of food and beverages; i.e., “recommended healthy foods” (*M* = 23.05, SD = 4.01, range 10–34), “inappropriate drinks and snacks” (*M* = 8.49, SD = 2.14, range 4–19), and “dairy products” (*M* = 11.94, SD = 2.84, range 4–22).

Correlational analyses were carried out to establish the direction and magnitude of the associations between the diverse consumption and diet related variables. There were no associations between “dietary” (measurement), and the aforementioned factors (subscales) with socio-demographics, as the parents' age, their gender and to what extent knowledge about healthy nutrition is sufficient for their children. Parents' type of education was found to correlate positively and significantly with “dietary” (*r* = .12, *p* < .05) and “recommended healthy foods” (*r* = .13, *p* < .01). Their children age was significantly negatively associated with “dietary” (*r* = −.19, *p* < .01) and “recommended healthy foods” (*r* = −.23, *p* < .01). Both aforementioned variables were not associated with the factors “inappropriate drinks and snacks” and “dairy products”. The average number of servings of *fruit* and serving of *vegetables* their child eats per day were found to correlate positively and significantly with “dietary” (*r* = .28, *p* < .01), same for both variables, and with “recommended healthy foods” (*r* = .36, *p* < .01) and (*r* = .50, *p* < .01), respectively. The variable “average number of servings of *vegetables* their child eats per day” was significantly negatively associated with “inappropriate drinks and snacks” (*r* = −.11, *p* < .05). The extent to which parents succeed in ensuring that their child eats healthily was found negatively and significantly associated with “dietary” (*r* = −.14, *p* < .01) and “recommended healthy foods” (*r* = −.36, *p* < .01), and positively and significantly with “inappropriate drinks and snacks” (*r* = .19, *p* < .01).

Oral hygiene behavior was found to correlate positively and significantly with the amount of fruit and vegetables the child eat (*r* = .11, *p* < .05) and (*r* = .23, *p* < .01), respectively, and negatively and significantly with the extent to which parents are successful in ensuring their child eats healthily (*r* = −.21, *p* < .01). No correlations were found between OHK and the 3 aforementioned variables.

In addition, correlational analyses showed the associations between the relevant oral health and diet or consumption related variables (Table 2). Only “recommended healthy foods” was significantly positively associated with OHB, and with OHK. “dietary” was found to correlate positively and significantly with OHB, and “inappropriate drinks and snacks” was found negatively and significantly correlated with OHB. Both were not associated with OHK. No correlations were found between “dairy products” and OHB or OHK.

**Table 2:** Intercorrelations (Pearson's) between the relevant main variables.

Variables	1	2	3	4	5	6
1. Dietary	–					
2. Recommended healthy foods	.73**	–				
3. Inappropriate drinks and snacks	.42**	-.05	–			
4. Dairy products	.69**	.22**	.17**	–		
5. Oral Health Knowledge	.51	.11*	−.05	−.02	–	
6. Oral Hygiene Behavior	.15**	.29**	−.12*	.01	.16**	–

* *p* < .05.

** *p* < .01.

Finally, a linear hierarchical regression analysis was performed to examine the multivariate relationships with oral hygiene behavior (OHB) as outcome variable. Conceptually, the extent to which parents succeed in ensuring their child eats healthily can be considered a predictor, while the amount of fruit and vegetables a child eats is better viewed as a measure of the amount of healthy food consumed. Therefore, the amount of fruit and vegetables a child eats were not included as predictors in the regression analysis, despite both being significantly associated with OHB. The variables that showed a significant correlation with OHB, namely “dietary”, “recommended healthy foods”, “inappropriate drinks and snacks” and “the extent to which parents are successful in ensuring their child eats healthily” were included as predictors in this analysis. All variables were entered at once.

The regression analysis showed that “recommended healthy foods” and the extent to which parents are successful in ensuring their child eats healthily were significant independent predictors of OHB [R^2^ = .10, F(4,415) = 12.20, *p* < .01]. In this model, the variables “dietary” and “inappropriate drinks and snacks” had no significant effects. This model proved to be significant and accounted for 10% of the variance in OHB (Table 3).

**Table 3:** Hierarchical regression of ‘recommended healthy foods’ and the extent to which parents are successful in ensuring their child eats healthily on oral hygiene behavior (OHB) in the total sample (*N* = 420), controlling for s1.

Variables	Oral hygiene behavior			
	*β*	s1	*t*	*p*
‘Recommended healthy foods’	.28	.044	3.26	.001
‘Dietary’	−.05	.029	−.54	ns
‘Inappropriate drinks and snacks’	−.07	.059	−1.08	ns
The extent to which parents are successful in ensuring their child eats healthily	−.11	.150	−2.09	.037

## Discussion

Since the relationship between nutrition and oral health is a broad concept, and accurately estimating the relative impact of diet or consumption and oral hygiene behavior on oral health is essential for developing adequate and tailored nutritional and oral health interventions (26), the first phase of this cross-sectional study consisted of the development of a valid and reliable dietary scale or consumption subscales specific related to OHB. Instead of using a large number of items, it is most adequate and feasible to use subscales, i.e., “recommended healthy foods”, “inappropriate drinks and snacks”, and “dairy products”. These subscales are not only statistically, but also theoretically adequate, and in contrast to the 17-items *dietary measurement*, the “recommended healthy foods” (7-items) and “the extent to which parents are successful in ensuring their child eats healthily” were independent predictors of OHB.

Previous Lithuanian studies, one on nutrition and the other on oral health, have shown that parents with higher educational types were more likely to mention the quality of food choices than parents with only primary education (14). It was found that parents had very good knowledge of their children's oral health, and that parents with higher education not only had better oral health knowledge but also generally exercised more parental control over their children's oral hygiene, with a clear correlation between oral health knowledge and oral hygiene (10). In this study, parental oral health knowledge was found to be correlated only with “recommended healthy foods”, while parental education type was associated with “dietary” and “recommended healthy foods”. The observed associations between “recommended healthy foods”, “dietary” and OHB, assessed using the modified OHB index from parents’ perspective (10), were correlational and do not imply causation. And just as OHK and OHB were correlated, this did not lead to an improvement in OHB (10); this also applies to knowing which healthy foods are recommended, it does not mean that it will lead a better or optimal OHB.

This study used non-probabilistic sampling, which may have introduced self-selection bias, as parents interested in their child's health were more likely to participate. Distribution through school channels may have excluded less involved parents. The anonymous, self-completed design increased the risk of social desirability and recall bias. Furthermore, 101 responses were excluded due to an error, meaning that something went wrong technically or procedurally, or ineligibility, because the respondent did not meet the requirements or criteria for the activity in question. This could potentially distort the results if the excluded participants differed systematically from one another.

Moreover, this study has several potential limitations. First, the self-report nature of a questionnaire, despite the use of Lithuanian validated indexes and scales (10, 14, 22, 23), may have introduced social desirability bias. This could lead parents, even though they are generally highly educated, to provide answers that may not accurately reflect their true opinions and diet and consumption behaviors. It is also a limitation that the responses are from the perspective of the parents and not specifically from their children. Nevertheless, this is typical of all self-report studies anyway, and these findings suggest that parents had no or less problem reporting socially undesirable and inappropriate drinking and snacking behavior.

Various terminology is used in the literature and because the 17-item “scale” (a series of questions) is taken from the cross-sectional Lithuanian COSI study (14, 23), description has been used and interpreted here: “Dietary” or diet refers to the types and amounts of food and drink a person regularly consumes, and consumption habits refers to the use or consumption of food and drink to satisfy needs or desires.

Although it is unclear what the Cronbach's *α* and Gutmann Lambdas 2 of this used dietary “scale” and its derived subscales is, the lower internal consistency of the subscales in this study is sometimes just below the generally accepted threshold for acceptable reliability. Therefore, the obtained scores should be interpreted with caution, as measurement error can attenuate the observed associations and reduce statistical power. This may indicate sample-specific characteristics or self-selection bias, and suggests the need for further refinement or validation of the subscales in future research. It should be noted that the existing indices of the previously used questionnaire were designed to indicate actual or reported OHB and OHK from the perspective of parents, and therefore neither is considered a scale. Calculating reliability (Cronbach's *α*) to check the internal consistency of the items is therefore not very meaningful and unnecessary (10).

Furthermore, it is possible that the sample of parents in this study, conducted in the city of Kaunas, is not representative of the entire parent population. The sample would also have been different if parents and their children lived in rural areas or small villages outside Kaunas. Furthermore, parents who were less interested in or concerned about their privacy and anonymity may have been excluded from the recruitment process.

In summary, these results suggest that the “recommended healthy foods,” “inappropriate beverages and snacks,” and “dairy products” subscales may be useful for identifying dietary patterns related to oral health behaviors, and that the used modified OHB index can be considered an adequate approach from the behavioral and oral health sciences (10, 22, 24). The findings can also contribute to needs assessments, nutrition and dietary management, including oral health education, and the tailoring of preventive measures for parents and their preschool- and elementary school-aged children, for public health and primary care professionals, nutritionists, dietitians, and oral health professionals.

Future research should incorporate other socio-psychological and/or cultural-context factors to investigate the effects and maintenance of recommended health food and consumption habits in relation to oral health behavior, and may be in (mal)nutrition related to oral discomfort (OD) scale (27). The process of preparing the OD scale in the Lithuanian language and culture should be carried out carefully (28, 29). Also in the development of tailored interventions focus could be more on changing parental attitudes and promoting positive text framing within persuasive communication (30, 31) for recommendations on healthy food that support optimal oral hygiene practices. Especially, these findings support a multidimensional assessment of dietary patterns in relation to OHB, highlighting “recommended healthy foods” as a key predictor and underscoring the importance of integrated, parent-based assessment in health promotion strategies. Furthermore, more diverse populations with a balanced age, gender and educational type distribution should be used to improve generalizability. Overall, this study contributes to a more nuanced understanding of the links between dietary and consumption habits and oral health. It points to areas for further research and practical application, without going beyond the available data. After all, caries is a multifactorial condition that can arise not only as a result of poor eating habits (32).

## Data Availability

The original contributions presented in the study are included in the article/Supplementary Material, further inquiries can be directed to the corresponding author/s.
